# Membrane Applications in Autologous Cell Therapy

**DOI:** 10.3390/membranes12121182

**Published:** 2022-11-24

**Authors:** Risto Martin, Rui Lei, Yida Zeng, Jiachen Zhu, Hong Chang, Hua Ye, Zhanfeng Cui

**Affiliations:** 1 Department of Engineering Science, Institute of Biomedical Engineering, University of Oxford, Oxford OX3 7DQ, UK; 2 Oxford Suzhou Centre for Advanced Research (OSCAR), University of Oxford, Suzhou 215123, China

**Keywords:** autologous cell therapy, bioreactor, hollow fibre membrane bioreactor (HFBR), cell culture

## Abstract

Stem cell and cell therapies, particularly autologous cell therapies, are becoming a common practice. However, in order for these technologies to achieve wide-scale clinical application, the prohibitively high cost associated with these therapies must be addressed through creative engineering. Membranes can be a disruptive technology to reshape the bioprocessing and manufacture of cellular products and significantly reduce the cost of autologous cell therapies. Examples of successful membrane applications include expansions of CAR-T cells, various human stem cells, and production of extracellular vesicles (EVs) using hollow fibre membrane bioreactors. Novel membranes with tailored functions and surface properties and novel membrane modules that can accommodate the changing needs for surface area and transport properties are to be developed to fulfil this key role.

## 1. Introduction

Through the manipulation, expansion, processing, and subsequent re-implantation of a patient’s own cells, autologous cell therapies are now used in the treatment of otherwise incurable diseases and degenerative conditions. As the cells used in these therapies are derived from the patient themselves, complications associated with immunological rejection may be negated. Consequently, autologous therapies are considered to be more readily achievable than other technologies in the field of regenerative medicine. To date, successful autologous cell therapies in clinical practice include:

- Autologous chondrocyte implantation (ACI) for cartilage repair [1].
- Autologous cell therapy for treatment of burns [2],
- Autologous stem cell transplantation for the treatment of multiple myeloma and multiple scoliosis [3], and
- Chimeric antigen receptor T-Cell (CAR-T) therapy for the treatment of blood cancers [4].

**Figure 1:**
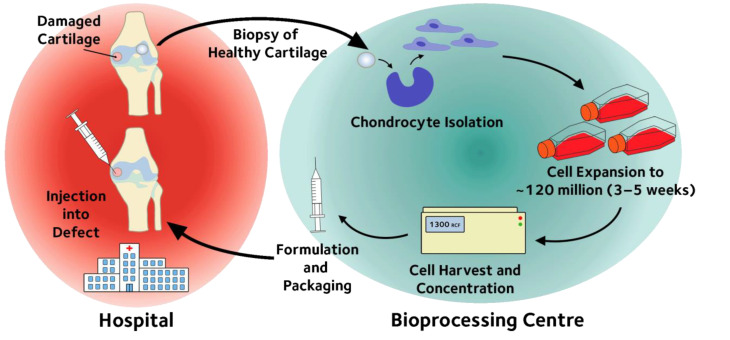
The processing steps of the approved ACI procedures based on the commercial process [5].

Autologous cell therapies, such as chondrocyte implantation therapy, are multi-step processes [5] as shown in the block flow diagram in Figure 1. In such a process, cells are initially obtained from the patient either through biopsy or a blood sample. Consequently, in contrast to traditional therapies, each treatment is bespoke to an individual patient. Processes developed for the production of autologous therapies are therefore not based on “scale up”, but rather on the parallel “scale out” using single use disposable platforms.

**Figure 2:**
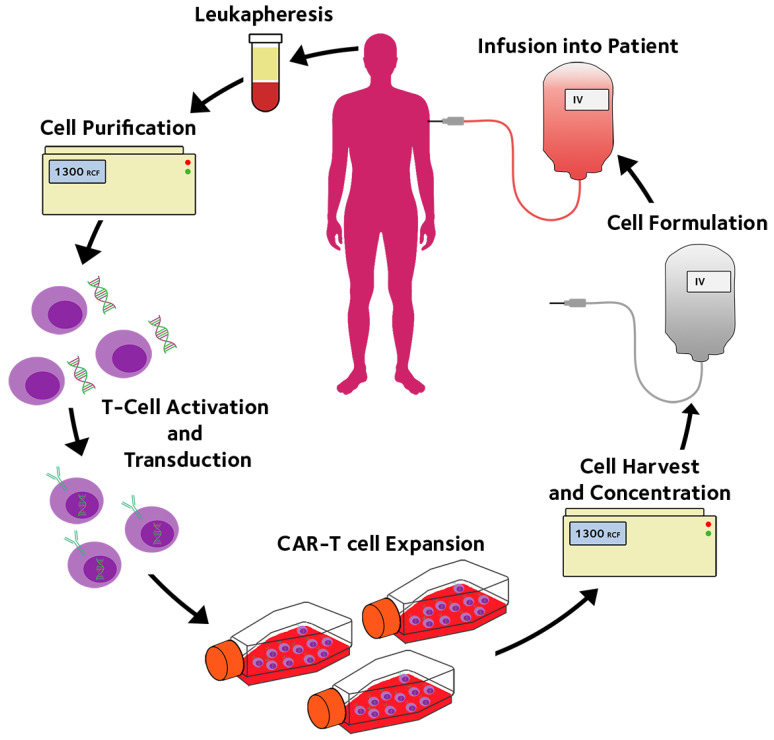
Simplified schematic of the processing steps in CAR-T manufacture for immunotherapy of cancers [8].

One of the main challenges to autologous cell therapies is their high cost. For example, CAR-T therapies approved for use by the NHS in the United Kingdom, tisagenlecleucel or Kymriah, come with the steep price tag of GBP 282,000 per treatment, with studies revealing the real cost being even higher when considering post-treatment care [6]. Bioprocessing and manufacture of the cellular products account for a large fraction of the cost of goods. Current processing technologies are largely based on mimicking laboratory cell manipulation and culture with different degrees of automation [7] as shown in the process flow diagram Figure 2. Infrastructure investment to create ultraclean environments (good manufacture practice, or GMP) is necessary for the “open operation” where the product or component are exposed to the operational environment. There is an urgent need for novel bioprocessing technologies, new equipment, and new devices, to form a fully enclosed, fully automatic, and single use system, to perform all the processing steps in hospitals without the need for dedicated GMP facilities. In this way, membranes can play an important role.

Membranes offer a physical barrier (excluding liquid membranes) with selective transport properties and different surface properties. With the desirable properties, membranes can be used to solve some key issues, in a genius way, for the bioprocessing and manufacture of autologous cellular products. Herein, we review the application of membrane technologies towards, CAR-T therapy, the culture of adherent cell types, expansion of stem cells, and isolation of extracellular vesicles (EVs).

## 2. Hollow Fibre Membrane Bioreactors for CAR-T Immunotherapy

It has long been understood that the immune system plays an essential role in cancer development and growth [9], constant “immunosurveillance” by the immune system seeking and eradicating potential cancer cells [10]. Further studies [11] demonstrated that specific T lymphocyte populations, killer T cells, are heavily involved in this natural cancer prevention through the hunting down and killing precancerous and cancerous cells [12]. These T cells have an innate “guidance system” in the form of T Cell Receptors (TCRs), which they use to recognize cancerous and precancerous cells [13]. These TCRs are sensitive enough to distinguish single amino acid changes in antigenic peptide sequences [14]. However, to avoid the development of autoimmunity, it is estimated that more than 90% of all immature T cells and all the T cells with self-reacting TCRs are destroyed before they reach maturity [15]. Thus, most T cells in the blood stream are nonreactive or only weakly reactive against mutated self-antigens on tumours. It is necessary to circumvent this problem to effectively use T cells as a form of cancer therapy.

The first form of T cell therapy utilised Tumour-Infiltrating Lymphocytes (TILs) that are found inside tumour tissues [16]. The rational for this was that T cells inside tumour will have a higher chance of reacting with mutated self-antigens on tumours. However, in spite of this TIL therapy has enjoyed only limited success [17].

A breakthrough in the effectiveness of this therapy was achieved by combining the low-reactivity high-cytotoxicity T cell therapy with Monoclonal antibody (mAb) therapy, which on its own has high reactivity but low cytotoxicity. This was achieved through genetic modification of T cells, enabling researchers to attach high affinity cancer specific antibodies called Chimeric Antigen Receptors (CAR) onto the surface of highly cytotoxic T cells to create the first CAR-T cells [18]. Through multiple clinical trials, it was demonstrated that these newly created CAR-T cells were exceptionally good at treating certain types of cancer [19]. In particular, the anti-CD19 CAR-T cell therapy for the treatment of B-cell lymphoma had been extremely successful [20] and subsequently six therapies have been commercially approved by the FDA including, ABECMA (idecabtagene vicleucel, Bristol Myers Squibb, New York, NY, USA), CARVYKTITM (ciltacabtagene autoleucel, Janssen Biotech, Inc., Horsham, PA, USA), TECARTUSTM (brexucabtagene autoleucel, Kite Pharma, Inc., Los Angeles, CA, USA), Kymria™ (tisagenlecleucel, Novatis, Basel, Switzerland), Yescarta™ (axicabtagene ciloleucel, Kite Pharma, Inc., Los Angeles, CA, USA), and Breyanzi (lisocabtagene maraleucel, Bristol Myers Squibb, New York, NY, USA) [21,22,23,24,25,26].

A key technical step in CAR-T therapy is to transduce and expand the CAR-T cells to about 1 billion cells for a couple of treatments. This is usually done using traditional cell culture flasks or wave-bag bioreactors. It should be pointed out the wave-bags are made of gas permeable membranes to allow gas exchanges during the culture. CAR-T therapy had been immensely successful in clinical trials and was generally regarded as “ground-breaking”. However, its clinical implementation and uptake has been slow [27] and has been plagued by problems such as the high cost and risk of associated side effects [28,29]. CAR-T cell expansion has been identified as an important challenge to improve the efficiency, uniformity, and controllability of the cell products.

As illustrated in Figure 2, all the currently approved CAR-T therapies use autologous peripheral blood or apheresis as the cell source [21,22,23,24,25,26]. Traditionally in pharmaceutical production cost is reduced through economy of scale during scale up. By using bigger bioreactors and producing bigger batches, the unit cost of the product can be lowered [30]. However, the need for autologous cells means that doses must be made-to-order as opposed to made-to-stock, which ultimately leads to higher unit cost. Not only does this increase the patient-to-patient curative effect variation [31] but also limits the scale of production. Thus, CAR-T manufacture capacity may only be increased by scaling out, increasing capacity by adding more equivalently functional production units, rather than scaling up [8]. Consequently, most CAR-T manufacturers still use largely manual flask or bag-based culture systems, these flask or bag based culture systems can be easily scaled out by adding more flasks or bags. However manual flask or bag-based cultures require large centralized GMP facilities with a lot of support equipment and space to maintain a sterile environment, which makes them very space inefficient and more important costly.

**Figure 3:**
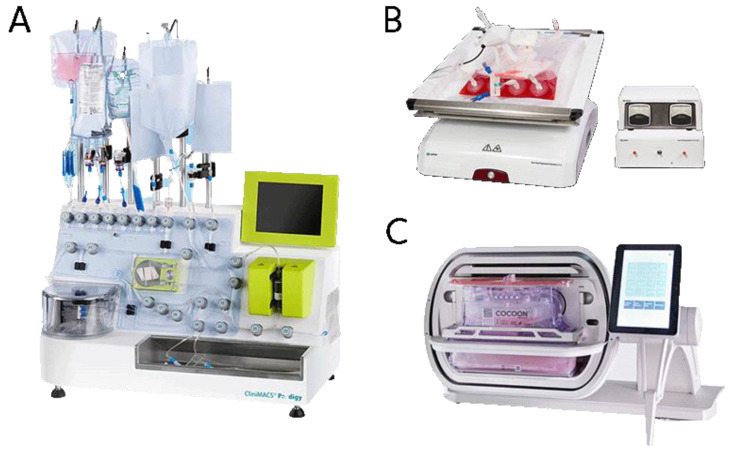
The (**A**) CliniMACS Prodigy, (**B**) Xuri cell, and (**C**) Cocoon, standalone closed bioreactor systems, edited from [36,37,38].

To negate these issues, standalone closed bioreactor units have been developed to replace flask or bag-based cultures, examples of which may be seen in Figure 3 and a comparison of which may be seen in Table 1. Several clinical CAR-T trials have used systems such as CliniMACS Prodigy from Miltenyi Biotec for their production [32,33,34], which provides an end-to-end platform for the expansion of both adherent and suspension cell types. The Cocoon platform from Lonza is another similar system. Each of these platforms is supplied with a touch screen interface and software which enables protocol design, online process monitoring and logging. The Xuri cell expansion system [35], which is based on the WAVE bioreactor platform, uses a large culture bag which is sufficient to expand large numbers of CAR-T cells with semi-feed of fresh medium. None of these systems can maintain ‘chemostat’ and uniform environment for the cultured cells [34].

**Figure 4:**
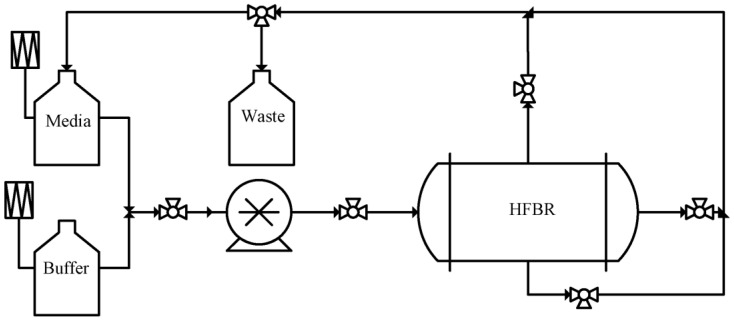
Block flow diagram of a typical hollow fibre bioreactor configuration consisting of a positive displacement pump, hollow fibre cartridge, media, buffer, and waste reservoirs.

Hollow fibre membrane bioreactors (HFBRs), with continuous perfusion, can precisely control the extracellular environment and control the cell culture process. Since their first development in 1972, HFBRs have been touted as a means to overcome many of the issues presented by vessel and wave bag based platforms, by offering a closed system with automated temperature, gas concentration, and inlet media flow rate control [39]. Typically consisting of a controller, positive displacement pump, HFBR cartridge, waste, media, and buffer reservoirs, connected in a continuous loop [40], as illustrated by Figure 4, HFBRs effectively facilitate the removal of waste products while simultaneously providing fresh nutrients directly to the cells, which ultimately allows for the support a much higher cell densities [41,42,43]. Membranes used in HFBR cartridges are typically produced through immersion precipitation phase inversion in which a polymer solution is extruded through a spinneret into a precipitation bath, in which asymmetric hollow fibre are formed with a pore density gradient in the radial direction, before being rinsed and stored [44]. Polymers commonly used for cartridge production include, Polyvinylidene fluoride, Polysulfone, and cellulose [45].

Depending on the cell type and the intended product, HFBRs may be configured such that cells are grown in the lumen of the membranes or in the extra capillary space (ECS) in between the hollow fibres (HFs). Suspended cells, such as CAR-T cells, are ideally grown in the ECS, in which a near fluid shear stress-free environment is maintained. In this configuration, high cell numbers may be maintained as membrane bound advective mass transfer facilitates the maintenance of high transmembrane concentration gradients for efficient nutrient transfer. Cells may also be grown in the HF lumen, while being exposed to fluid shear forces to induce mechanical stimuli. The use of membrane bioreactors for cell culture and bioprocessing is not new, and the advantages in retaining cells while removing secreted products and metabolic wastes have been explored for a wide range of bioprocessing applications in both research and commercial settings.

HFBRs can be made into an automatic system with single use functionally closed cell culture consumable set, such as the Quantum Cell Expansion System. Studies have shown that the Quantum Cell Expansion System can produce large quantities of functional T-cells at clinical dosage levels [46] with increased potency [47].

Chang et al. developed a single use modular hollow fibre membrane bioreactor system, specifically for CAR-T cell expansion. The system is fully closed, fully automated, and integrated with all necessary steps involved including T cells transduction and activation, expansion, product formulation, volume reduction and final harvest. It might be possible that such a system can be placed at the hospital bedside without GMP bioprocessing facilities and cell transportation, reducing the cost of CAR-T cell processing significantly.

The membrane provides a physical barrier to enable a fully closed system. This minimises risk of contamination. As terminal sterilization techniques such as gamma radiation ethylene oxide or UV sterilization cannot be used on living cells, strict aseptic processing protocols must be followed to prevent contamination. When using traditional flask or bag-based cell manufacturing processes, due to the prevalence of open processes involved there is a higher microorganism exposure risk [48,49]. This is because, when using static flask-based cell cultures or rocking motion-based bag cell cultures, these processes involve multiple open processes such as, adding media or cell harvesting [50,51].

Traditional culture platforms in contrast to HFBR systems are very manual labour intensive. This in turn increases the costs associated with the operation of such processes due to the reliance on a large highly skilled labour force. In addition to the costs this also increases the chances of human error and batch variability, a phenomena realised in the increased rate of failure rate of commercial CAR-T products compared to other pharmaceutical products [52].

An outstanding advantage of the HFBR system developed by Chang et al. is to integrate all processing steps into the fully closed and automated system, with T cells genetically modified so that they express the chimeric antigen receptor, activated to unlock their proliferation potential, expanded, concentrated, formulated before being infused back to the patient [52,53]. Without the HFBR system, these multiple step manipulations mandate the use of multiple pieces of equipment for each step and technician’s operations which is very labour intensive and introduces potential batch to batch variability and risk of contamination.

## 3. Membrane Bioreactor for Autologous Cell Expansion

Autologous cell therapies, either using primary cells or various stem cells, involve expansion of the target cells from biopsy to a large number of cells (millions to billions) of desired quality and functions. Initial protocols use manual operations following the procedures in the laboratory research. Bioreactors for cell expansion soon appeared as a key requirement in autologous cell therapy. While multiplate, spheroid, stirred tank and packed bed bioreactor systems go some way in achieving this goal, the dependency of these systems on labour intensive tissue culture practices and low cell yields inhibits their wide spread commercial success [54].

Since their first use in their expansion of human choriocarcinoma cells, HFBRs have been used to culture many adherent cell types, for research and clinical applications, including, but not exclusively, endothelial cells (ECs), satellite cells, leukaemia cells, stromal cells, human cord blood derived mononuclear cells, and erythroid cells.

ECs were first cultured in the lumen of HF membranes in 1995 [55]. It was noted that when exposed to hydrodynamic shear stress that endothelial cells developed a phenotype more akin to that found in vivo and may even explain the heterogeneity of ECs throughout the body [56]. Through the compartmentalisation of cells grown in the lumen and ECS, HFBRs have been used to recapitulate more complex cellular models. By co-culturing of ECs with various other cell types, models have been developed for the blood brain barrier [57], and leukemic lymphocytic metastasis.

HFBRs have shown great promise for stem cell expansion. Autologous stem cells, including mesenchymal (MSCs) or hematopoietic stem cells (HSCs), as well as induced pluripotent stem cells (iPSCs), have enormous medical potential owing to their potential to differentiate into specific cell linages [56]. HFBRs have been demonstrated as a means by which iPSCs may be expanded, similar to embryonic stem cells [57,58,59], although it was noted that further process refinement was still required [60]. HFBRs have also shown promise [60,61] in culturing MSC, the stem cells more readily for clinical applications. While shear stress may be used to induce differentiation of MSCs, when expanding MSCs the minimisation of shear stress is desirable, and to this end HFBRs excel [58,62]. Consequently, HFBRs have been used for MSC expansion in large scale automated systems for a wide range of derived tissues including neural [63,64], skeletal [65], and for immunomodulatory effect [66].

HFBRs proved effective in producing red blood cells. It is known that the hematopoietic cellular niche plays an integral role in the regulation of haematopoiesis. Owing to their rigid mechanical properties and the relatively hydrodynamic shear stress free environment in the ECS, HFBRs have gained attention as a platform which may support ex-vivo haematopoiesis. Through the co-culture of human stromal cell line (HS-5) and human cord blood derived mononuclear cells a population of CD 34+ progenitor cells were expanded in serum free conditions. Further examination revealed the deposition of ECM proteins between the membranes, thus demonstrating the three-dimensional (3D) nature of HFBR cultures [67]. This promising result garnered much attention, alluding to the potential of ex-vivo red cell production. Subsequently a tertiary polyurethane scaffold was employed in the ECS to mimic the structure of trabecular bone. Following the seeding of umbilical cord blood mononuclear cells, hematopoietic populations were supported for 28 days allowing for the continuous harvest of enucleated red cells in serum free conditions [68,69]. Consequently, HFBRs offer a means by which red blood cells may be produced ex-vivo at greatly reduced cost and potentially alleviate the dependency of donations [70].

The clear need for HFBRs for regenerative medicine has naturally drawn great attention from industry and various commercial systems have been developed. For example, TERUMO, fibreCellSystems, The CellCultureCompany, and Oxford Mestar now offer a range HFBR solutions for bioprocessing applications [45,71,72,73,74]. Each of these companies has targeted slightly different markets with; TERUMO providing a wholistic end to end cell culture solution for the expansion of cells in the form of the Quantum platform. With full control of media flow rate, temperature and gas compositions afforded by the automatically fed cabinet, the Quantum system facilitates reduced manual operations thereby reducing the chance of contamination while improving reproducibility [73]. To date the platform has been used for, the expansion of CD3+ T-cell [75,76], MSCs [46,77], adipose stromal cells [78], neural stem cells [54], and iPSCs [79]. The DUET pump platform developed by FibreCellSystems is intended for laboratory scale HFBR systems. Depending on the intended application a range of different membrane modules may be used, each produced with a range of membrane materials and pore sizes. It is the relatively low cost and flexibility, compared to complete cell expansion systems, associated with the FibreCellSystems platform that make it so attractive to researchers. The DUET platform has consequently be used in the investigation of immune regulatory effect of MSCs [66], for the production of EVs at a GMP standard [80], the interaction of ECs and myeloid leukaemia [81], and even in the investigation of stem cell health in microgravity conditions onboard the international space station [82]. The CellCultureCompany has produced several platforms suitable for a wide range of applications and scales of operation. At the smallest scale, intended for use in a standard tissue culture incubator, the HF Primer system is most suitable for research applications. Increasing in size the AutovaxID is a standalone system intended as a pilot scale platform to bridge the gap between R&D and production scale. Finally, the AcuSyst-Maximizer and AcuSyst-Xcellerator provide a scale-up pathway intended for production scale applications of between 500–2000 L fed-bed equivalent in size, while fitting in a cabinet the size of a refrigerator. The RegenMed Solution system developed by Oxford Mestar is a modular system integrating cell seeding, expansion, harvest, and final formulation together, and has proved successful in CAR-T culture and human MSC expansion.

## 4. Membrane applications in EV Production

Membrane technology can play an important role in extracellular vesicle (EV) production. EVs are a type of lipid bilayer membrane-bound nanovesicles secreted by cells. EVs produced by stem cells [83,84,85,86,87] are an important secretory product that might be responsible for various therapeutic effects of stem cell therapy [88,89,90], and hence can be an important therapeutic product for anti-aging as an example. However, research and application of this potential cellular secretome product is largely hindered by its low productivity and the challenges of isolation and purification.

As EVs are secreted products of cells, the yield of EV products is partially dependent on the ability of cell expansion [91]. Current production of EVs is mainly based on traditional flask-based cell culture processes, which is largely limited by scale, highly manual labour-intensive and time consuming, while also being associated with high chances of human error and batch variability. As previously discussed HFBRs may yield large numbers of adherent stem cells in a small 3D space, and consequently, may be used to increase the yield of EVs. A key practical advantage that HFBRs systems offer is that they may also function as a part of the downstream process. Owing to the semi-permeable nature of the membranes in an HFBR, membranes with suitable pore size allow the mass transfer of nutrients and waste through continuous perfusion while retaining EVs that are produced. Through this means, EV products can accumulate and be preliminarily concentrated within the cell culture compartment of an HFBR. This in turn is beneficial for downstream purification processes as the smaller start volume makes subsequent liquid handling easier [92]. Recently, Mendt et al. demonstrated the Terumo Quantum platform may be used as an effective platform for the consistent expansion of exosomes, a type of EVs. The exosomes produced in turn were shown to increase the rate of survival from pancreatic cancer [93]. Furthermore, Williams et al. and Potter et al. demonstrated that MSC-derived exosomes could be successfully obtained from Quantum cell expansion system, while preserving their therapeutic effects [94,95]. Similarly, Cobin et al. demonstrated that EVs could be produced efficiently and reproducibly with MSCs derived from multiple human donors with a HFBR platform from FibreCellSystems, while preserving the phenotype and functionality of the cells [80]. It should be noted that EVs reflect the state of the cells from which they originate as their content can be considered as a fingerprint of the type and status of their parent cells. Thus, in order to ensure that the EVs harvested from ana HFBR are of desired type, quality, and function, it is vital to continuously monitor the status of the cells in the bioreactor as previously mentioned. Currently, sterile sampling of metabolic products and macromolecular biomarkers is readily achievable to monitor the cell condition through the use of membrane probes. However, it is very challenging to achieve direct online monitoring of the status and functions of the parent cells, and there is currently no existing method that would allow direct characterization of EVs within the bioreactors during the cell culture, due to the limitation of current characterization method. Therefore, it is also essential to characterize both the phenotype of their parent cells after the culture in the bioreactor and the isolated EVs sample itself in terms of size, morphology, expression of EV markers and functional potency, prior to the next stage research and application.

EVs are nanoparticles with a size range mainly from 30–1000 nm. They are usually spherical, carrying cargoes including proteins, RNA species, DNAs, and lipids. Several common methods are available for EV isolation and purification, the pros and cons of which may be seen in Table 2. Among these EV isolation methods, ultracentrifugation, immunoaffinity capture, precipitation, and size exclusion chromatography have limitations of poor scalability, high cost, introduce unwanted agents and requiring extra isolation steps, respectively [90]. In contrast, membrane filtration is capable for high throughput, is more readily scalable, requires less capital investment for equipment, is faster, and less labour intensive. Consequently, ultrafiltration (UF) is a very promising EV separation method for large-scale manufacture of EVs intended for clinical applications.

Microfiltration (MF) and UF can readily be used for EV isolation and purification. MF using membranes with pore sizes ranging between 0.1–0.8 µm is normally used to remove larger particles from the target EV fraction [96]. Track-etched polycarbonate membranes with pore sizes from 30–600 nm have been utilized for fast EV isolation or detection in specialized filtration devices, e.g., a cyclic tangential flow filtration (TFF) system [97], a TFF based microfluidic chip [98], an integrated double-filtration microfluidic device [99], an exosome total isolation chip [100], a lab-on-a-disc integrated with two nanofilters [101]. Commercial centrifugal ultrafiltration filters with multiple membrane materials and molecular weight cut-off (MWCO) ranging from 10–300 kDa are used for volume reduction of a large amount of biofluids and conditioned medium from cell culture before EV isolation [102], or as an additional concentration step for the relatively dilute EVs after other isolation steps, for example, size exclusion chromatography (SEC) [103]. It was reported by Vergauwen et al. that the most popular membrane type for EV concentration is regenerated cellulose with MWCO of 100 kDa, based on the records in the EV-TRACK knowledgebase [104].

UF-TFF can also be applied to EV isolation and purification. In TFF with a suitable choice of membrane MWCO, EVs larger than the pore sizes can be retained, concentrated and recirculated in the capillary space where biofluid or conditioned cell culture media is continuously pumped in, while smaller molecules, such as some small proteins, salts, and solvents can travel through the pores on the membrane and be removed in the permeate [105,106]. In addition, to increase the purity of EV products, diafiltration can be operated in TFF systems. Small molecules are further removed, buffer exchanged, or lowered by adding fresh exchange buffer at the same rate as the permeate flow rate. Compared to conventional batch-wise dead-end filtration, TFF is superior as it reduces the formation of filter cake and can operate large-scale EV isolation and purification in a continuous process. Choi et al. successfully isolated EVs from human adipose-derived stem cells by TFF with a 500 kDa MWCO membrane filter capsule, confirming the functional recovery in photo-damaged human dermal fibroblasts after EV treatments [107]. Higher purity might be obtained by combining different techniques with UF, although UF alone is applicable for EV isolation and purification [108]. EVs were reported to have been concentrated 50 fold through TFF with a 100 kDa MWCO membrane, followed by purification using chromatography column and further concentrated by 100 kDa MWCO centrifugal filter [109]. Finally, Hydrostatic filtration dialysis, a filtration technique which consists of filtration, concentration and dialysis using hydrostatic pressure of the fluid, has also been employed to isolate EVs from urine using dialysis membranes with 1000 kDa MWCO [110].

## 5. Needs for Research

Membrane bioreactors have an important role to play. When developing a platform for the production of cells or cellular products, such as with EVs, there are crucial considerations, such as, process conditions, regulatory approval, cost of device, quality control, membrane area, and product recovery. First, it is vital to establish uniform bioreactor conditions. This is necessary to ensure a homogeneous and reproducible product as cells are sensitive to the local environment. This is particularly important to the culture of stem cells as they are more susceptible to environmental perturbations.

There is always a need for new membranes that are biocompatible for single use medical devices. Unlike other industrial applications of membranes, the cellular product will be implanted or injected directly into the human body. Consequently, any material that is in contact with the product stream such as the membrane, housing case, potting material or glue must be approved for use in medical devices and produced in from pyrogen-free materials [111]. Regulatory issues should not be overlooked during the research and development of new membranes and modules which provide inevitable hurdles in the path to commercialisation. To prevent complications associated with cleaning, systems are commonly developed to be single use [112]. Consequently, the cost of such membrane device must be low, e.g., at 10 USD per square meter of membrane area or lower. Finally, the device will have to be pre-sterilised, usually with gamma radiation. This adds an additional selection criteria that needs to be considered when selecting membrane and housing materials [113].

Membrane biosensors may find a wider use in cell therapy. To meet regulatory requirements, it is necessary to have robust methods to monitor the process for quality control purposes. Currently, the sterile sampling of metabolic products and macromolecular biomarkers is readily achievable through the use of membrane probes and various sampling techniques like microdialysis [114]. In contrast, cell function can only be assessed with biomarkers and complicated assays. It is therefore challenging to achieve the online monitoring of cell function. However, as human cells do not grow quickly, offline assays offer a practical means of measurement although their use increases the risk of contamination. In addition, the direct characterization and quality control method for the production of cellular products such as EVs is still at its infancy. Specialized biosensors that can be integrated to membrane bioreactors are therefore required.

Novel membrane modules with changing and adjustable membrane areas are needed. Throughout the culture period of adhering cells if the area is insufficient, cell growth will be inhibited by contact inhibition. However, if the area is too large initially, the cell density on the surface will be low and the cells will not proliferate. This is also true for culturing suspended cells with an optimal cell density in the membrane reactor. The development of a membrane bioreactor which may increase the area of membrane exposed throughout culture, to maintain exponential cell growth, is therefore required.

Upon culture completion, the cellular product must be harvested, concentrated, resuspended, and formulated for cryopreservation and clinical application. Usually, the cell suspension is reduced to a volume that fits into a syringe for administration. Current protocols for harvest and formulation rely on labour- intensive multi-step processes. As with the culture process, through the use of an HFBR platform, it is possible to replace these manual steps with a membrane device capable of buffer exchange via dialysis, concentration, final formulation, and volume reduction. Such a device would be a closed system and may be fully automized, thereby greatly reducing the risk of contamination.

Single use and low cost membrane oxygenator for gas–liquid oxygen exchange is welcomed. Further to the use of membranes in bioreactors, membrane technologies may be used to optimize bioprocessing more generally. Membrane oxygenators have been widely used in the culture of shear sensitive cells and microbes, which was stemmed out from Extracorporeal Membrane Oxygenation (ECMO) [115]. The challenges with membrane oxygenators are primarily the cost of the device and suitability as a medical device. In addition, the use of an oxygenator increases the liquid holdup and should therefore be minimized as the culture media are expensive.

Cell separation using membranes to replace flow cytometry would be a big step forward. Currently, upon culture completion cell sorting and purification for stem and T-cell production is achieved through flow cytometry. The development of a flow through device for pre-treatment or cell sorting would represent a huge increase in efficiency. Such a device would likely be based on the difference in the surface properties of cells, and therefore operate upon affinity instead of size difference. Another application for affinity or ion exchange membranes would be the removal of macromolecules secreted by the cells, or from lysed cells, which otherwise negatively affect cell growth. The designing of such a membrane will need detailed understanding on the specific proteins produced by the particular cell type.

The separation and purification of EVs using membranes have great potential. Although membrane-based techniques are widely used for EV isolation, the interaction between EVs and membranes with different parameters, such as MWCO, materials, charged or not, and any kind of modifications, are rarely investigated to increase the selectivity and reduce the losses from EV binding to membranes. The process of filtration should also be studied and optimized to increase the efficiency and yield of EV isolation. Deformation, disruption, or loss of properties and function of EVs caused during membrane separation requires further investigation [96]. Additionally, while there are several commercial membrane-based separation products available, these have been originally developed for different processes and subsequently adapted to EV separations. Specialized membranes for EV isolation and purification based on the properties of EVs and EV-membrane interaction research are therefore required. Integration of stem cell expansion and EV production would increase the EV productivity enormously as the cells can be retained and cultured for a longer period of time while EVs are still produced.

## 6. Conclusions

As the age of personalized medicine dawns, many engineering challenges come with the realization of new technologies. For these technologies to reach full fruition, these challenges must be overcome. No technology better exemplifies this than CAR-T therapies. While, broadly considered to be ground-breaking, CAR-T therapies have failed to reach widespread clinical application, predominantly due to their prohibitively expensive cost per treatment. To date, the cost of pharmaceuticals has been minimized through the economy of scales. However, the bespoke nature of personalized medicine prevents this approach. Instead, cost effective scale-out, rather than scale-up, methods are imperative for success, as is an alternative business model.

Autologous cell therapies mandate the growth if a patient own cells for re-implantation back to the patient. Traditional flask and wave bag culture techniques adopted in scale-out processes are costly, labour intensive, and prone to contamination. HFBRs, developed for the culture of various cell types in both research and commercial settings, may offer an automated, cost-effective, closed system alternative. However, to implement HFBRs into a clinical setting, rigorous regulatory approval of commercial devices, quality control, and validation methods are necessary to ensure success, as there normally is not a second chance.

Membrane technologies represent a relatively untapped market in bioprocessing of autologous cells and cellular products. There are some interesting and challenging problems such as cell sorting and EV purification that provides an exciting research opportunity through the development of specialized affinity membranes. New membrane modules, e.g., those with adjustable membrane areas, would be interesting to develop too.

## Figures and Tables

**Table 1:** Comparison of platforms developed for the manufacturing CAR-T cell therapies.

Name	Company	Principle	Volume/Surface Area	Scalability	Gene Editing?	Temperature Control	Final formulation
CliniMACS Prodigy	Miltenyi Biotec	CentriCultUnit or External culture vessel	low/dependant on external vessel size	low	In place	Requires external temperature control when external vessel used. No reagent temperature control	Fill and finish capable
Cocoon	Lonza	Customizable cassette	460 mL—low	medium	In place	Duel environmental control for reagents and cell growth	Fill and finish capable
Xuri cell	Cytiva	Wavebag	0.3–25 L—medium	medium	In place	Integrated tray heater and sensors	External finishing required
Duet Pump	FibreCellSystems	Hollow fibre membrane	80 cm^2^–1.2 m^2^	medium	No	Requires CO_2_ Incubator	External finishing required
Quantum	TERUMO	Hollow fibre membrane	1.7–2.1 m^2^	High	No	Continuous control of temperature	External finishing required
HF Primer	CellCultureCompany	Hollow fibre membrane	1.5 m^2^	medium	No	Requires CO_2_ Incubator	Concentrates harvest but external finishing required
AutovaxID	CellCultureCompany	Hollow fibre membrane	80–100 L equivalent	High	No	Automated control of temperature	Integrated refrigerator for continuous harvest
AcuSyst-Maximizer	CellCultureCompany	Hollow fibre membrane	80–200 L equivalent	High	No	Automated control of temperature	Integrated refrigerator for continuous harvest and In-line filter for harvest clarification to reduce downstream processing
AcuSyst-Xcellerator	CellCultureCompany	Hollow fibre membrane	500–2000 L equivalent	High	No	Automated control of temperature	Integrated refrigerator for continuous harvest and In-line filter for harvest clarification to reduce downstream processing

**Table 2:** Pros and cons of common extracellular vesicle isolation methods.

Method	Principle	Throughput	Scalability	Cost	Operation	Effects on EVs
Ultracentrifugation	Sequential centrifugation step, separated EVs based on size and density	Large	Low	High equipment cost	Manual labour intensive, time intensive, batch variability	Mechanical damage
Immunoaffinity	Capture EVs based on their surface markers	Low	Medium	High cost for antibodies	Require a pre-concentration step, time consuming	Reversible step required
Precipitation	Use precipitating agent to induce the pelleting of EVs	Low	Medium	Medium/Low	Further purification required to remove the precipitating agents	Introduction of synthetic precipitating agents to EVs
Size exclusion chromatography	Separated EVs based on size with a packed column of with fine, porous beads	Medium	Medium	Medium	Require concentration step before and after	Minimal detrimental effects on EVs
Membrane filtration	Separated EVs based on size with filters	Large	High	Medium/Low	Time-efficient	Less detrimental effects on EVs

## Abbreviations

3D	Three-dimensional
ACI	Autologous Chondrocyte Implantation
CAR	Chimeric Antigen Receptors
CAR-T	Chimeric Antigen Receptor T Cell
ECMO	Extracorporeal Membrane Oxygenation
ECS	Extra Capillary Space
ECs	Endothelial Cells
EVs	Extracellular Vesicles
GMP	Good Manufacture Practice
HFMBs	Hollow Fibre Membrane Bioreactors
HFs	Hollow Fibres
HSCs	Hematopoietic Stem Cells
IPSCs	Induced Pluripotent Stem Cells
mAb	Monoclonal Antibody
MF	Microfiltration
MSCs	Mesenchymal Stem Cells
MWCO	Molecular Weight Cut-Off
SEC	Size Exclusion Chromatography
TCRs	T Cell Receptors
TFF	Tangential Flow Filtration
TILs	Tumour-Infiltrating Lymphocytes
UF	Ultrafiltration
